# Supplementary material for: Examining the exposure-reception-retention link in realistic communication environments via VR and eye-tracking: The VR billboard paradigm
Source: PLoS One. 2023 Nov 30;18(11):e0291924. doi: 10.1371/journal.pone.0291924 (PMC10688884; doi:10.1371/journal.pone.0291924)
Supplement: Attachment — Submitted filename: ResponseToReviews.docx [file pone.0291924.s001.docx]

***Response to reviews***

***Reviewer #1:***

Thank you very much for the positive feedback and for the time and effort spent in reviewing our manuscript! Below, we will respond to your comments in a point-by-point fashion, with excerpts of our changes to the manuscript in blue text^[1]^.

**R1.1. I like the idea of combined effort in using VR and Eye tracking. But I have a hard time understanding the manuscript. Frankly, I often second-guessed what the authors were trying to communicate. Simple English is preferred.**

We were happy to hear that you like the general idea of combining VR and eye tracking to study exposure to messages. We have revised the manuscript (including several proofings by native speakers) and streamlined the core argument.

Changes made: We revised the introduction. Edits are too voluminous to be copied here, but if you re-read the introduction and discussion sections, you will see that we took your feedback to heart.

**R1.2. The literature review only talks about how great the idea of combined usage of VR and Eyetracking is. Still, there is no reason for the experimental manipulation in the methodology. It seems that manipulating “free viewing” and “counting trash” is related to cognitive load. Authors may use the Limited Capacity Model of Motivated Mediated Message Processing (LC4MP) theory for reasoning.**

Thank you for this comment. We have updated the relevant section and included references that support our reasoning. This is also something that R2 pointed out, so please also note our response to R2.

*Introduction: …* To experimentally demonstrate the potential of this paradigm, we instructed half of the participants to count the trash placed alongside the highway. The other half was instructed to look freely while driving down the highway. It is well documented that such a parallel, attention-consuming task will distract participants (Cowan, 1998; Long et al., 2018; Neisser & Becklen, 1975) and should consequently lead to fewer gaze fixations on the billboard messages (Eckstein, 2011; Han & Kim, 2004). This simple manipulation also helps to connect the new VR billboard paradigm, which is primarily concerned with objectively assessing message exposure and its consequences for incidental memory, with the broader literature on message processing in which comparable tasks were often used (Huskey et al., 2020; Lang, 2000; Madore et al., 2020).

**R1.3. Please also justify why the experiment deliberately had half of the billboards be “health-related topics” and others be “typical advertisements.” Does “health-related topics” mean public service ad (PSA)?**

Yes, the billboards about health-related topics can be described as PSAs. We have clarified this and thank you for the comment. Regarding our motivation to include PSAs and commercial ads, there are two related reasons: First, given that US billboards often feature health-related messages, we deemed it important to include these topics. Second, we are personally more interested in effective health messaging than advertising and will focus more on health-related materials in our upcoming studies (albeit we will still have to include commercial advertisements). We did not have a specific hypothesis about memory for PSAs vs. commercial (and thus report the comparison as an exploratory analysis).

*Methods:* … Billboard messages: We developed 20 visual billboard messages using templates from Canva.com. Half of the billboards were about health-related topics (e.g., drinking, vaping, smoking, marijuana, seatbelt use, and distracted driving), resembling typical public service announcements that characterize outdoor billboard advertising in the U.S.. The second half of the billboards were typical advertisements (e.g., retail, lawyer services, hotels, and restaurants/food). The billboard messages all featured basic imagery along with some text, and their design was deliberately kept relatively simple but still typical of the kinds of billboards present on U.S. highways (see Fig. 1 and online repository at github.com/nomcomm/vr_billboard_paradigm).

*Discussion:* … Likewise, our experimental messages are also limited in variety, number, or design- and content elements. For instance, we deliberately designed our billboard messages to feature no-name brands (e.g., lawyers, hotels, and nearby restaurants) as well as health-related public service announcements because these make up a large share of billboard advertising.

*Discussion:* … Lastly, we also explored the degree of memory for health-related vs. commercial billboards, finding no significant differences. Nominally, health-related billboards were slightly more often recalled (also see Fig. 6), but the effect was insignificant (F(1,36) = 3.53, p = 0.07). Across both conditions (independent participants), the same billboards tended to be recalled more often, as indicated by a significant vector correlation between trash counting and free-viewing (r = 0.82, p < 0.001).

**R1.4. And I think the experiment is trying to test something related to distance, as subjects need to provide feedback on spatial presence. But I don’t follow the meaning “the distance between successive billboards was assigned randomly and then kept fixed across participants.”**

We apologize for the misunderstanding. We have clarified the corresponding section in the revision. The situation is as follows: The billboard stands are lined up along the highway. We could have simply placed them at - say 500, 1000, 1500, 2000 meters from the origin. However, this would create a very monotonous environment. Hence, we chose to position the billboard stands in “jittered” fashion (e.g., 500, 1150, 1670, 2302 meters, etc.). The inter-billboard distances were chosen to be large enough to not cause distraction from each other (as in an attentional blink experiment, Shapiro, 1997). We have clarified this in the revision.

*Methods:* …We added a sunny, blue sky with a few clouds as well as trash objects (empty soda cans - used as targets for the trash-counting task). The twenty virtual billboard signs were placed along the road using 3D-billboard model stands, and the billboard messages were assigned to each of the billboard stands in a programmatic fashion (randomized across participants). The billboards were placed along the roadside semi-randomly, with the constraint that the inter-billboard distance had to be large enough to prevent interference (Raymond et al., 1992).

**R1.5 And authors should be careful in claiming the realism of VR. Not all VR looks like reality. It all depends on the production. Most VRs I have seen for experiments are poorly made due to the production cost. I can’t access the stimuli in this study. But if the authors want to justify how real it looks, it is worthwhile to elaborate more about it. For example, does the cloud in the sky (seen in the screenshot) move and vary in shape? Besides the billboard, is the surrounding environment changing with that 10mins simulation (say, another car passed by, more grass, a hut appeared somewhere)? And what’s the virtual car speed? Are the brands featured on all billboards in fictitious names?**

You are raising an important comment: We agree that it is easy to get *some* results in VR studies rather quickly, but that it takes more effort to produce high-quality environments. We discuss this point in the revision and - as you suggest - we elaborate further on the details of the environment.

To give you an impression of the quality achieved in our highway environment, we can refer you to the actual model: [https://sketchfab.com/3d-models/highway-50-near-cold-springs-18ebd36db2e748a9bb0e 88ee73006041](https://sketchfab.com/3d-models/highway-50-near-cold-springs-18ebd36db2e748a9bb0e88ee73006041). Sketchfab allows you to access at least a screen version of the Highway 50 stretch near Cold Springs and if you have a VR headset, you can also inspect it virtually. As you can see, the environment is captured via high-quality photogrammetry. Also, you can see that the grass landscape changes, but there are no huts and no other buildings (as this is a direct copy of the actual Highway 50, this matches reality). Your next question was about the sky. We added the sky by placing a so-called sky dome over the highway model. The size of this model was adjusted so that the sky merged seamlessly with the horizon. Regarding your question about clouds: The clouds moved as the participants drove along the highway, i.e., as they changed their position with respect to the sky dome. However, the clouds did not move as would happen in stormy weather conditions; this was actually a conscious decision we made because the weather conditions in Nevada tend to be rather dry, fairly sunny, and stable. If we wanted, we could animate them. Finally, we opted to keep the simulation/experiment simple by not having opposing traffic. Indeed, from our own road trips, we can attest that it is quite common not to have opposing traffic in such an area for a while. But you are right in stating that it could be added. As for speed, we allowed a speed of up to around 55 mph; in general, what happened was that participants accelerated up to the limit and kept driving down the highway, as if in cruise mode. In sum, as you can see, the environment is relatively realistic compared to the comic-style VR simulations. Also, several participants commented very favorably about the degree of realism it achieved. While we do not claim that our environment was 100% realistic (e.g., there is no breeze), the basic visuals were of relatively high quality, and participants commented favorably about the experience.

*Introduction:* … First, VR can create increasingly realistic environments - whether it is riding a rollercoaster, walking along a virtual plank, or driving down a highway. Visual information is particularly central to the human mind/brain and is one of the primary channels of human information intake that VR can virtually simulate. Thus, researchers can design virtual environments that mimic the essential appearance of a wide variety of human visual environments. Of course, current VR applications are not perfect depictions of reality (e.g., they lack olfaction, haptics, and even visual depictions may not be perfectly detailed), but VR has proven to be a useful tool for examining a wide array of perceptual, cognitive, and socio-emotional mechanisms (Blascovich et al., 2002; Parsons, 2015).

*Methods:* …VR highway environment and billboard signs: We developed a virtual highway in which 20 billboards were placed along the road. The core highway model was downloaded from Sketchfab.com and consisted of a digitized 3D photogrammetric model by the Nevada DOT (Highway 50 - Near Cold Springs - Download Free 3D Model by NDOT, 2018). It featured a straight stretch of Highway 50 taken near Cold Springs with arid and desert-like vegetation alongside. We added a sunny, blue sky with a few clouds as well as trash objects (empty soda cans - used as targets for the trash-counting task).

*Discussion:* … Like all research, the current study has several limitations. One limitation is that although the VR experience featured a version of a real highway drive (a digital twin of Highway 50 near Cold Springs), some elements of real life were missing (e.g., opposing traffic, birds, curves, and passages through towns, etc.). Although driving on relatively empty highways is not uncommon, it is clear that adding these variables would make the simulation more realistic and generalizable.

**R1.6 Measurements should be clearly stated. Is the frequency of fixation or the duration of fixation being used for analysis? Or both? And it is unclear how the relationship between fixation and recall/recognition data is calculated. Is it code “1” for billboards being fixated and recalled/recognized and code “0” for others? Or is there some variation in the fixation measurement based on the number of times or duration? Also, are “spatial presence” and “occurrence of symptoms” open questions?**

Thank you for the feedback. We have worked to clarify the specific measurements. In brief, perhaps the most central variable in this study was - as you correctly state - whether (1) or not (0) a billboard was fixated. Note that this variable is determined for every participant (because everyone’s viewing behavior differs and the billboards were assigned randomly from participant to participant). As a secondary measure (beyond this dichotomous “1-vs.-0” fixation status) we also examined the fixation count. This has also been clarified.

With regard to the spatial presence and VR symptoms questions, these were based on the spatial presence scale and the VR sickness questionnaire, respectively. The previous version included a citation to these sources, but the numbered citation style made this difficult to understand. In response to your comment, we have thus expanded on the description of these self-report measures.

*Methods:* … The main variable measured during the virtual drive was whether participants fixated on a given billboard. Fixations were detected algorithmically and saved to disk in a spreadsheet together with the fixated billboard’s name. The fixation threshold was set to 0.25s. Thus, for every participant, the virtual drive yielded a spreadsheet containing where a given billboard was fixated (and how often, e.g., time 15s, fixation, billboard_1, etc.). Because billboard images were randomly assigned to individual billboard sign positions, a python program was written to resort the individual images to a given participant’s eye-tracking data (e.g., time 15s, billboard_1, drunk_driving.jpg, …), allowing for subsequent data aggregation across participants and billboard messages.

The recall data (information on whether a participant brought up the billboard during the free recall task, e.g., “I recall seeing a billboard about drunk driving”) was merged with the fixation information, and so was the recognition data (information on whether a participant recognized the billboard at the end of the study from a list of billboard images at the end of the study).

In the analysis, we first compared the overall number of fixations as well as the memory performance between conditions (free viewing vs. trash counting). These analyses were carried out to examine the overall effects of the condition (free viewing vs. trash counting) on viewing behavior (fixation count) as well as memory performance (measured via recall and recognition).

Next, the main central analysis compared message recall between the experimental conditions, zooming in specifically on how viewing a billboard vs. ignoring it relates to memory and how this interacts with the instructed viewing condition. To this end, we used a mixed ANOVA with the factors Fixation Status (coding whether a message was looked at, 1 vs. not looked at, 0) and Viewing Condition (free viewing vs. trash counting). Note that the factor Fixation Status varies within subjects varies within subjects because every participant passed all 20 billboards and looked at some vs. ignored others. Viewing Condition represents a between subjects factor. In addition to computing this analysis for the recall data, we also ran the same analysis on the recognition data.

Methods: … Specifically, we asked participants to report on their experience of spatial presence (Hartmann et al., 2016) and the occurrence of symptoms while in VR (VRSQ survey, (H. K. Kim et al., 2018).

**R1.7 ANOVA is a statistical test to analyze the difference between the means of more than two groups. As far as I understand, the analysis compares two groups: free viewing vs. trash counting. Or is it indeed a multiple-group analysis? The statistical analysis should clearly state what is being compared.**

We have updated the manuscript to remove ambiguity regarding the statistical procedure used. You are correct in stating that the current study used ANOVA to compare the means of the DV (memory: recall) across conditions in a 2*2 design. It seems that our previous description failed to convey this clearly. Specifically, the free-viewing vs. trash-counting condition represents one factor, but the other factor is the *fixation status*, i.e., whether a billboard was looked at or not. In our view, this factor is the more central and certainly the more innovative one, whereas free-viewing vs. trash-counting was only introduced in order to link our results to previous studies and to gain experimental control over the viewing behavior (i.e., impact the fixations).

Thus, the factor *fixation status* varies within subjects (billboards looked at vs. not looked at), whereas the free viewing vs. trash counting represents a between-subjects comparison. This creates 4 cells (2*2) and the DV (memory: recall as the primary measure) is measured for each of these cells. Thus, a mixed (within-between) ANOVA is the appropriate test. We have updated the manuscript to make this more clear.

*Methods:* … Next, the main central analysis compared message recall between the experimental conditions, zooming in specifically on how viewing a billboard vs. ignoring it relates to memory and how this interacts with the instructed viewing condition. To this end, we used a mixed ANOVA with the factors Fixation Status (coding whether a message was looked at, 1 vs. not looked at, 0) and Viewing Condition (free viewing vs. trash counting). Note that the factor Fixation Status varies within subjects varies within subjects because every participant passed all 20 billboards and looked at some vs. ignored others. Viewing Condition represents a between subjects factor. In addition to computing this analysis for the recall data, we also ran the same analysis on the recognition data.

We document the analysis and provide code in the study’s online repository at github.com/nomcomm/vr_billboard_paradigm.

**R1.8. The dose-response relationship is about the correlation between the effect and the volume of the same dose. Since the experiment has not repeatedly shown the same billboard, there is no dose-response relationship. It is not appropriate to claim a causal pathway of the dose-response relationship.**

We have toned this down, removing the term dose-response. However, please note that what you describe about the ‘correlation between the effect and the volume’ is not inconsistent with the current study and its results: You are right in saying that the experiment has not repeatedly shown the same billboard. Instead, the billboards were always there. Critically, however, peoples’ viewing intensity varied (in a self-determined fashion): Some people may never look at a given billboard (like swallowing zero pills), others may look at it once (swallowing one pill), and others may look at the same billboard repeatedly (like taking in the information three times). Empirically we find that the more people look at the billboard (the more information they take in through their senses), the higher the likelihood they will recall it. To further spell out the analogy: In pharmacological testing, it also doesn’t really matter if one puts 5 pills on the table (opportunity for exposure) or 3, but what matters is the quantity that people swallow (actual exposure). The latter is what the present paradigm allows us to measure. We have revised the paper to discuss this.

*Discussion:* … These results all support our main argument, which is that exposure and reception are the prerequisites of message retention (memory). The present approach thus has value for pinpointing the mechanism that leads from exposure to retention: Specifically, the causal chain starts with the presence of a message in the information environment (opportunity for exposure), then the person noticing and taking in the message (actual exposure, reception), to subsequent memory (retention). Further evidence for this is also provided by the marked differences between fixations and memory in the trash-counting vs. free-viewing conditions, and by the fact that messages that were looked at more were recalled more often.

**R1.9 I would strongly advise authors to have thorough proofreading the manuscript. References 2 and 11 are missing at the back. There is no figure label at the top or bottom of the figures. Do check on the grammar. For example, “If true (see, e.g., the rebranding of the social media company FaceBook to Meta), VR might be on the way to becoming a messaging environment in and of itself.”**

Thank you for this feedback. The manuscript has been proofread by a native speaker. We apologize for the omission of the references. Of note, this happened after the original manuscript (which contained all clean references) was returned to us because we had placed the figures and their captions in line with the text (which we still view as more readable, albeit not APA-compliant). This error got introduced in the reformatting process, and we apologize for it.

**Reviewer #2:**

Thank you very much for the overall positive feedback and for the time and effort spent reviewing our manuscript! Below, we will respond to your comments in a point-by-point fashion, with excerpts of our changes to the manuscript in blue text.

**R2.1. This claim "...we know shockingly little about how many messages we encounter in our daily lives" on p.5 is inconsistent with a point you make earlier in the manuscript. A variety of data sources (e.g., Nielsen data) give us good indicators about opportunities for exposure. Can you clarify?**

Thank you for pointing this out. This was ambiguously worded. It has now been clarified, toned down, and the “shockingly little” language has been removed. Indeed, Nielsen data and similar sources give us ample information about opportunities for exposure. However, at the end of the day, such aggregate metrics all require a leap of faith whether viewers actually had their eyes on the screen. We clarify this by further emphasizing the distinction between “opportunities” and “actual exposure”.

*Introduction (also see other changes in the introduction):*

**Exposure as the Cornerstone of Message Effects and Marco- vs. Micro-Level Measures** (new heading and following paragraph)

Exposure is key to media and message effects (Hornik 2002; Slater 2004; Shoemaker and Reese 1990; Vorderer and Reinecke 2012). It is obvious that if audiences are not exposed to a message, communication can not have any effect - just like a pill not swallowed cannot have any pharmacological effect. This applies to all kinds of messages - from advertisements in newspapers and on the web, commercials on TV or radio, or billboards along the road. Given the central role of exposure as a prerequisite of message effects, virtually all media track metrics like audience size or reach (de Vreese & Neijens, 2016; Smit & Neijens, 2011), and there is solid evidence that these relate to outcomes like brand or ad recall (Slavtchev & Wyrwich, 2023; Tellis, 2003; Hornik, 2002; Slater, 2004; Southwell et al., 2002). However, strictly speaking, such approaches only quantify “opportunities for exposure.” To keep within the pill analogy, this is like knowing how many pills were delivered or bought by consumers (opportunity to be exposed), but not whether an individual also took the pill (actual exposure).

Thus, to determine whether a person actually notices and subsequently processes a message, we need to take a micro-level approach. Zooming in on this nexus between exposure and reception is challenging because it requires novel measures and must be done on an individual-by-individual and message-by-message basis. However, doing so seems necessary because otherwise, information from media ratings remains detached from the psychological reception processes, i.e., how the message enters the brain from the communication environment.

*Introduction:* … To summarize, exposure is the basis of message effects. If we want to delve into the processes and mechanisms of information reception and retention, we must take a micro-level perspective and measure exposure as it occurs (Potter, 2008; Southwell et al., 2002). Furthermore, both exposure and memory should be studied in a more incidental and ecological manner. This is more representative of how messages are encountered and processed, but these conditions are difficult to realize. As a result, we know a lot about processes that occur post-exposure ( Goldstein, 2014; Huskey et al., 2020), but these steps occur after the initial act of looking at a message. In other words, our understanding of the sequence from media content to reception processes to subsequent effects suffers from a measurement gap at a very early stage.

**R1.2. On p.5, you write "our knowledge and theories about exposure and its transmission into message effects are woefully incomplete." Is "woefully incomplete" really the current state of our knowledge? Visual attention studies are not the only way of measuring message exposure and plenty of work has been done examining exposure to and subsequent recall of auditory messages. It seems you overstate the novelty of the project. Even in the visual domain, using VR, there is work looking at exposure and recall (e.g., https://doi.org/10.1080/15252019.2020.1846642). Of course, that work has not used eye-tracking (as you have), but others have used eye tracking in increasingly naturalistic advertising contexts (e.g., work by Claire Segijn). A more thorough review of the literature, and tempering of your claims, is warranted.**

**R1.3. Related, some theoretical starting points exist for thinking about the problem. For instance, Lang's LC4MP provides a decent foundation for thinking about exposure -> recall as you are interested in here. I'm not saying you have to use or cite Lang or the LC4MP. But I don't think we're at ground zero when it comes to theorizing about the issue.**

[Please note that we took the liberty to merge these two points in order to address them jointly]

We thank you for this feedback, which made us realize that this was also poorly worded and seems to have led to a wrong impression about our goals. We have rewritten this to be less ambiguous and hint to existing work, including the references/authors you mention.

Given that both your comments (see below) as well as those of reviewer 1 focus more on the variable *viewing condition* (free-viewing vs. trash-counting) than on *fixation status* (whether a billboard was looked at or not), it seems that our original introduction was not effective in motivating a focus on this variable. In our view, the inclusion of this represents the main innovation - as opposed to the more common ‘dual-task’-style manipulation. In fact, we added the viewing-condition comparison only to demonstrate that our paradigm can capture this well-known effect; moreover, it also gives us an ‘experimental grip’ to influence participants’ fixations. As said, we have worked to further clarify this, which is probably the most central aspect of our entire revision^[2]^.

Again, we want to emphasize that it was not our intention to characterize the state of the field as “we know nothing about any of this.” Instead, as you point out, we know quite a bit about message processing, which is the focus of entire theories (such as the LC4MP). Importantly though, it was not our goal to test any specific message-processing theory with this study. Rather, what the challenged sentence (‘woefully incomplete’) tried to allude to was what is known in memory research as the famous ecological memory debate (Neisser, 1978). This debate centered around the importance of studying phenomena in ecological and representative contexts (see e.g., Neisser, 1997; Brunswik, 1935, or Miller et al. 2019). We admit that this was not optimally worded, but the first part of the sentence (*“to the extent that these situations are the natural norm rather than the exception, our knowledge ...”*) contains an important qualification. With this in mind, we see the main contribution of this work in the ability to ascertain *“actual exposure” as it occurs and to study how it leads to incidental memory.* This bridges the gap between i) social-science studies that often study exposure in the aggregate (and without measuring actual exposure, but only opportunities) and ii) laboratory studies of message processing in which exposure typically plays no role because message exposure is “forced onto participants.” Regarding the latter, you are of course right that existing work (now cited) studies advertising in “ increasingly naturalistic contexts”. However, a majority of research (including ours and including the present study) still suffers in important ways when it comes to studying exposure and the subsequent memory formation in a more natural manner. Thus, as simple as the question we examined here is (i.e., how much do we retain from merely looking at a message?), it has to the best of our knowledge not been investigated in this way, and particularly not using objective eye-tracking measures.

Finally, with regard to your comment about the LC4MP, we absolutely agree LC4MP provides a solid framework for the viewing condition task manipulation (free-viewing vs. trash counting). As indicated above, it was not our intention to question this, and we provided relevant citations (albeit not to the LC4MP, but to more basic work; after all, although the LC4MP is a fine model, it is more an adaptation to the messaging context and not a theory of the underlying mechanisms, such as attention, memory, or motivation. Part of this misunderstanding is perhaps due to the numbered citation format used by PLOSOne, which makes it difficult to integrate source studies: Specifically, (old) references 10 and 11 (there was an error in ref 11 apparently - see above) refer to seminar articles and textbooks on memory research; others point to seminal work in cognitive psychology and communication about memory and exposure (refs 1-12). For instance, you can see already in the original version of the paper (ref 10) that cite what we see as the foundational literature for this kind of work: the early 1970s Craik & Tulving/Craik & Lockhart studies that are cited in every textbook on memory. This is also the base citation in A. Lang’s article on the LC4MP, which adapted these ideas from cognitive psychology to communication. The experiments by Craik and colleagues are relevant to our study because they deal directly with incidental memory (although they didn’t have the kind of “visual search”/”visual attention” component that characterizes our study – more on this below - see R1.5)^[3]^. Because both reviewers bring up the LC4MP, we have added a reference to the introduction.

*Introduction:* … Although most of our memories are formed while we are engaged in everyday activities, much memory research focuses on more deliberative memory tasks and examines memory formation under laboratory conditions. This approach has led to important insights, but critics have long demanded that memory be studied under ecological conditions that focus more on everyday memory (Hasson et al., 2008; Neisser, 1982, 1988). The same is true for studies examining message processing, which are also largely conducted in experimental laboratory settings (Huskey et al., 2020; Lang, 2000).

However, several obstacles make more naturalistic research difficult. These include the challenge of studying peoples’ behavior in natural environments and manipulating experimental factors therein. This clearly applies to psychological memory research, but also to research specifically focusing on memory for messages as it is studied in communication and advertising (Beard et al., 2022; Coronel & Bucy, 2020; Dardis et al., 2018; S. Kim & Southwell, 2017; Rossiter & Percy, 2017; Segijn et al., 2017; Zeigler, 1970).

*Introduction: …* To experimentally demonstrate the potential of this paradigm, we instructed half of the participants to count the trash placed alongside the highway. The other half was instructed to look freely while driving down the highway. It is well documented that such a parallel, attention-consuming task will distract participants (Cowan, 1998; Long et al., 2018; Neisser & Becklen, 1975) and should consequently lead to fewer gaze fixations on the billboard messages (Eckstein, 2011; Han & Kim, 2004). This simple manipulation also helps to connect the new VR billboard paradigm, which is primarily concerned with objectively assessing message exposure and its consequences for incidental memory, with the broader literature on message processing in which comparable tasks were often used (Huskey et al., 2020; Lang, 2000; Madore et al., 2020).

Our main question was whether looking at individual billboards would predict subsequent memory. The abovementioned reasoning on the exposure-retention link predicts that a substantial portion of messages that were looked at (i.e., for which actual exposure occurred) should be committed to memory. In addition to this fundamental hypothesis, we expected to confirm basic effects about task competition, namely that the instruction to look out for trash should lead to less fixations on the billboards and in turn lower memory performance compared to the free viewing condition.

*Introduction:* … Eye-tracking is an obvious candidate for examining the exposure-reception nexus for visual messages. Eye-tracking provides direct information about where an individual is looking (Duchowski, 2017), which is in turn related to what messages a person is paying attention to. Due to these desirable characteristics, eye-tracking is widely used to study how individuals respond to messages, where and for how long they look, and so forth (Bix et al., 2009; Chen et al., 2017; Le Meur & Baccino, 2013; Lochbuehler et al., 2016; Segijn et al., 2021).

However, previous eye-tracking studies were also mostly conducted in controlled laboratory environments, and most eye-tracking research in the context of message reception was done using screen displays. This approach is perfectly valid for studying how people browse websites, read online reviews, or engage with other related modes of messaging (Coronel et al., 2023; Segijn et al., 2021), but it does not apply to other messaging environments, such as highways, streets, and other contexts in which we encounter messages that are not screen-based (Foulsham et al., 2011).

**R1.4. Can you provide a citation to back up this claim on p.8 "It is well documented that such a parallel, the attention-consuming task will distract participants and should lead to fewer fixations to the billboard messages."**

Thank you for this. You are right that a reference here is missing. This is now corrected.

*Introduction:* …It is well documented that such a parallel, attention-consuming task will distract participants (Cowan, 1998; Long et al., 2018; Neisser & Becklen, 1975) and should lead to fewer fixations to the billboard messages (Eckstein, 2011; Han & Kim, 2004). This simple manipulation also helps to connect the new VR billboard paradigm, which is primarily concerned with objectively assessing message exposure and its consequences for incidental memory, with the broader literature on message processing in which comparable tasks were often used (Huskey et al., 2020; Lang, 2000; Madore et al., 2020).

**R1.5. Memory research in highly controlled experimental media contexts shows moderate effect sizes for memory (https://doi.org/10.1080/23808985.2020.1839939) and more recent work shows that VR can actually dampen memory for messages, especially in instances of high spatial presence like your study (e.g., https://doi.org/10.1016/j.compedu.2022.104532). Why was a large effect size selected a priori and what is your justification? Please note, I do see that you estimate power at .95 and then round up. But how does it look with a smaller effect size?**

Thank you also for this comment and for the reference to the meta-analysis, which is now cited.

Your argument about moderate effects of memory in highly controlled contexts matches our experience gained from studying thousands of participants in cognitive psychology lab courses over many years. Based on these insights, we can confirm that effect sizes are moderate for advanced memory paradigms comparable to the ‘experimental media contexts’ you refer to. However, especially for classical memory paradigms the effects are exceptionally strong (e.g., for serial position curves; Murdock 1962, forgetting curves, Ebbinghaus, 1913, release from proactive interference, Wickens et al., 1963, or levels of processing, Craik & Lockhart, 1972) - so strong that they almost resemble psychophysics studies.

*Methods:* The sample size was set a priori to 40 participants, which was chosen based on power considerations. For instance, Neisser (1964) reported that unattended targets in visual search tasks were not remembered at all, and work on incidental memory suggests that even the shallowest encoding operations build some memory (Craik & Lockhart, 1972). Moreover, the viewing condition should impact strongly how much participants look at the billboards, leading to much more fixations in the free-viewing than in the trash-counting condition (Chen & Zelinsky, 2006). These aspects combined suggest that billboards that were looked at should be recalled markedly more often than those that were not. We determined that for an assumed large effect (d = 1.2), a sample size of 16 per group would be sufficient for high power (1-β = 0.95, α = 0.05) to detect a between-group difference in the number of recalled billboards. We rounded this number up to 20 per group.

With this in mind, please note that the critical difference that drives all effects is between billboards that were not looked at (and, as we argue, not even encoded) and the ones that were looked at^[4]^. This relates to classical work in cognitive psychology, which showed that non-target items in a visual search task leave no memory trace (e.g., Neisser, 1964). Similar arguments could also be derived from classical work by Postman, Broadbent, or the already mentioned Craik/Lockhart study (1972). Said differently, while many of the studies in the meta-analysis manipulated more advanced aspects of how encoding happens, this study manipulated^[5]^ whether it happens at all. If it does, then this boosts the likelihood that a billboard will be remembered by a factor of 5-20 (depending on the condition). These considerations led us to expect a strong effect, which we have now further clarified in the text.

Regarding your second comment about the potentially dampening effects of VR for memory: The quoted study refers to a comparison between a VR vs. non-VR context. Also, despite the title suggesting otherwise, there are actually no significant negative effects of VR on memory. Likewise, the billboard racing study by Wang & Yao, 2020 compared VR- vs. screen presentation and found no significant difference either. Importantly though, our study took place completely in VR, comparing the fate of items therein based on fixation status as a within-subjects variable. It would indeed be interesting to compare our VR results with those from a field study (e.g., with mobile glasses), and your comment prompted us to allude to this possibility in the discussion.

Discussion: … Of course, there would also be the possibility to use mobile eye-tracking tools during a highway drive to carry out a field study. …

**R1.6. For the recognition data, is there a reason you didn't calculate signal detection measures (https://doi.org/10.3758/BF03207704)? This would give more insight into the thing you are worried about (e.g., "gauge participants’ tendency to recognize all messages as seen" p.10). It would also give more insight into the psychological processes related to recognition. There is an imbalance between targets and foils (20 vs 4), but it should be possible to calculate signal detection measures, and there are some nice packages for doing this easily in R (e.g., https://rdrr.io/cran/psycho/man/dprime.html).**

Thank you for this comment as well! You are right that signal detection measures are the default option for the analysis of recognition data. The reason why we didn’t calculate them is as follows: If you look at the right panel of Figure 3, which assesses recognition rate as a function of *fixation status* (i.e., whether a billboard was looked at or not) and *condition* (free viewing vs. trash counting), we couldn’t figure out a way to calculate dprime for this analysis (and we didn’t find one in the literature). At a global level, we can compute a dprime for every participant (because from the post-hoc survey we have the number of hits and false alarms/false-recognitions). However, if we delve down into the conditions of fixation status, then we’d have to compute a dprime for the ‘looked at’ vs. ‘not looked at’ conditions separately. However, we only have one global false alarm rate (assessed by the number of falsely recognized distractor items at the end of the study); moreover, fixations to billboards vary across participants. As a result, we cannot combine the hit-information (which is computable) with the false-alarm rate (which only exists at the global level of the participant)^[6]^. This prompted us to compute the relatively straightforward recognition rate. Together with the finding that most participants exhibited no false alarms at all, and only very few participants exhibited some FAs, we believe this is the most direct way to report the results. Of note, this argument (about not being able to assign FAs to the individual condition) does not apply to the analysis in Fig. 2. For this analysis, we could compute and report dprime values. However, to be consistent and not confuse readers with switching axes, we opted to keep the y-axis the same across Fig. 2 and 3.

*Results:* Note that we do not report signal detection (SDT) analyses here because the upcoming analysis (see. Fig. 3) does not allow directly computing SDT measures and we want to keep metrics consistent across the subsections of the results. However, the findings also hold when running the overall recognition analysis with dprime values instead of the recognition rate.

**R1.7. Is it possible to get an F1 score your your machine learning model?**

Thank you! We now compute an F1 score in addition to the reported ROC-AUC score. The average weighted F1 score was 0.58. Of note, there is a heavy class imbalance because “no recall” is the default state, and recall is relatively rare. This is a challenge for machine learning models, and we thus used penalization.

*Results:* Using a 5-fold cross-validated SVC prediction, we found that this simple model performed well, with a ROC-AUC score of 72.8% compared to 50% for a dummy classifier (note that we used penalization to deal with the imbalance classes, i.e., recall being rarer than no recall; weighted F1 score: 0.58).

**R1.8 Minor comment: 1. Citation #11 is missing from the reference list.**

Thank you for catching this. There were some hiccups in the submission process, which ultimately led to some formatting issues, for which we apologize. This should now be corrected.

Thank you very much again for the valuable suggestions and helpful comments.

1. For easier reference, we provide the citations here in Author-Year-style, whereas citations in the main paper are numbered as per PlosOne guidelines.
2. Also: It is really a shame that there are not yet good options to demonstrate what this study “looks and feels like” in VR. We believe that the specific way in which our study strikes a balance between the advantages and disadvantages of laboratory experiments (high control, low realism) and field research (high realism, no control) is much better conveyed through experience than through description
3. For instance, Craik’s studies include manipulations of “encoding operations” designed to disentangle what one could call “mere exposure” from deeper semantic processing, and to study the influence of encoding time vs. encoding operations. At this stage of our research, we didn’t focus on this all, but it could be added easily.
4. A critic could of course argue that this is trivial because there can be no memory for a message to which a recipient was not exposed. However, this is actually the key point of our argument: If exposure so critical, how come that there is such a gulf between social scientific studies of messaging (aggregate level, ambiguous re: opportunities for exposure) and the controlled lab studies (forced exposure, but limited for real-world contexts beyond screen-based messaging).
5. Strictly speaking, we didn’t manipulate this - but the participants behaved such that they exposed themselves to messages or not - but the result is the same: some messages were looked at - others not.
6. One thing that could possibly be done to still compute a dprime is to assume that people could guess equally across the fixated/non-fixated billboards; then, one could assign the false alarms based on the proportion of fixated-vs.non-fixated billboards (which varies from participant to participant). However, this seems troublesome because it would assume that the criterion does not change across fixation conditions (which is implausible) and the assumptions of assigning the false-alerts proportionally is also problematic.
