# Supplementary material for: Examining the exposure-reception-retention link in realistic communication environments via VR and eye-tracking: The VR billboard paradigm
Source: PLoS One. 2023 Nov 30;18(11):e0291924. doi: 10.1371/journal.pone.0291924 (PMC10688884; doi:10.1371/journal.pone.0291924)
Supplement: Attachment — Submitted filename: R1 comment round 2.docx [file pone.0291924.s002.docx]

Thank you for providing the revised manuscript. The authors have made improvements that make it easier to understand their intended message.

After reviewing the manuscript, it appears that three main topics have emerged. However, the write-up lacks a comprehensive and coherent presentation, and it seems a bit scattered. I would suggest that the authors streamline one of the topics and provide a better narrative walk-through.

1. The combined use of VR and eyetracker: The literature review and discussion primarily focus on this topic. However, the write-up fails to establish a clear connection with the actual experiment, which is related to exposure and cognitive resources. Although the discussion covers the use of technology, it is not the main priority.

2. The importance of exposure measurement: The literature review does not provide much elucidation on this topic, and there is no theoretical framework presented. Surprisingly, this topic is introduced in the discussion section. It is unclear why the authors chose to compare fixations between the two experimental conditions instead of conducting a correlation or regression analysis to link the number of exposures to recall. Furthermore, please rephrase line 352, which states that billboards that are never looked at are practically never recalled. If subjects can recall a billboard without seeing it, it would be considered blind guessing.

3. The allocation of cognitive resources: Similar to the exposure measurement topic, there is no discussion or theoretical framework about this in the literature review section. The authors mention a 2x2 ANOVA analysis to clarify this topic, but it is unclear how the groups were divided for the fixation count. Were they divided into high and low groups? Additionally, the write-up mentions a resulting table in line 332, but I couldn't locate it.

To improve the manuscript, I recommend the authors focus on one topic and provide a clearer narrative throughout. They should also address the issues related to the importance of exposure measurement and the allocation of cognitive resources by providing a more comprehensive literature review and theoretical framework. Additionally, clarifying the analysis methods and providing the missing table would enhance the manuscript's clarity.
