# Supplementary material for: Examining the exposure-reception-retention link in realistic communication environments via VR and eye-tracking: The VR billboard paradigm
Source: PLoS One. 2023 Nov 30;18(11):e0291924. doi: 10.1371/journal.pone.0291924 (PMC10688884; doi:10.1371/journal.pone.0291924)
Supplement: Attachment — Submitted filename: ResponseToReviews_R2.docx [file pone.0291924.s003.docx]

***Response to reviews***

***Reviewer #1:***

Thank you very much for the feedback and for the time and effort spent reviewing our manuscript! Below, we will respond to your comments in a point-by-point fashion, again with excerpts of our changes to the manuscript in blue text. We first respond to your overall comment about the three main topics and the narrative (R1.1), then address your specific questions about analytic details.

**R1.1. Thank you for providing the revised manuscript. The authors have made improvements that make it easier to understand their intended message.**

**After reviewing the manuscript, it appears that three main topics have emerged. However, the write-up lacks a comprehensive and coherent presentation, and it seems a bit scattered. I would suggest that the authors streamline one of the topics and provide a better narrative walk-through.**

**1. The combined use of VR and eyetracker: … 2. The importance of exposure measurement: … 3. The allocation of cognitive resources: … To improve the manuscript, I recommend the authors focus on one topic and provide a clearer narrative throughout. They should also address the issues related to the importance of exposure measurement and the allocation of cognitive resources by providing a more comprehensive literature review and theoretical framework. …**

We are pleased to hear that the revision helped to make the paper easier to understand! The three topics you identify are indeed close to what we intended to say. Thank you also for the feedback that we should streamline this all - we agree. We have thus followed your suggestion and revised the way in which we introduce the theory behind and the importance of exposure measurement. We have also shortened the introduction how this relates methodologically to eye-tracking & VR and revised the introduction of what you call cognitive resources. This has helped streamline the paper and weave a conceptual through-line for both the introduction as well as discussion.

*Changes to the paper:* … We have extensively revised the introduction and discussion. Changes are too numerous and widespread to be listed here individually. Please refer to the paper to review these edits.

As you can see, we have substantially changed the introduction to focus more on the definition, measurement, and effects of exposure, providing additional reasoning and references about its theoretical relevance and the limitation of previous attempts to measure exposure. Given that exposure already featured prominently in our title and the overall goal of the study was to introduce a new way of studying actual exposure (rather than indirectly inferring it from macro-level data about opportunities), this was a natural choice. To better reflect this focus and not be distracted by methodological details (although they are critical because they enable the theoretical advance), we opted to restructure the title to:

*Revised Title*: Examining the Exposure-Reception-Retention Link in Realistic Communication Environments via VR and Eye-tracking: The VR Billboard Paradigm

We also rewrote the section about selective attention, providing further references and justification of the theoretical rationale. Please note that we come at this from a cognitive neuroscience perspective on selective attention, and we do not espouse or test resource or load theories. Rather, the attention/distraction manipulation we use in this study is a simple way to manipulate participant’s fixations (and highly successful in doing so, as the results demonstrate), but it has never been the goal of this study to test theories of load or contribute to the literature on load (cognitive or perceptual), resources, or capacity. But we want to be clear and avoid misunderstanding about this issue: there is no question that the concepts of selective attention and load/resources are closely related, and we thus extensively reference these bodies of research. However, our goal was to leverage a very well-known effect - that distraction reduces attention - to demonstrate that this impacts visual information acquisition behavior (fixations) and results in reduced memory, which it did. This was thus a way to demonstrate the paradigm’s prowess. Overall, the revised version has been streamlined to emphasize how the study promotes method-theory synergy by enabling to overcome a significant theoretical shortcoming in the literature about exposure (i.e., the fact that it has often been called ‘exposure’, but not actually measured).

**R1.2. … Additionally, clarifying the analysis methods and providing the missing table would enhance the manuscript's clarity.**

Thank you very much for pointing this out. Below, we have pulled together your suggestions about analysis methods and addressed each one directly to ensure we thoroughly address all of the individual questions you raised.

**R1.2a The authors mention a 2x2 ANOVA analysis …, but it is unclear how the groups were divided for the fixation count. Were they divided into high and low groups?**

We apologize that this issue was still not as clear as it should be. The 2-factors for the ANOVA were i) viewing condition (free-viewing vs. trash-counting) and ii) fixation status (looked at vs. not-looked-at). The first factor is a group variable, i.e., participants there were assigned to either the free-viewing or trash-counting group a-priori (20 participants per group). The second factor, and likely the one you were unclear about, is the factor fixation status. This factor was defined individually based on participants’ viewing behavior, i.e., depending on whether and which billboards they fixated.

We understand that this analysis, in which variables are defined by each individual participant’s behavior, may not be as familiar as the conventional variable definitions based on stimulus identity, but the logic is quite simple: Imagine you were passing 20 billboards on a highway. We might find that you fixated on 12 billboards and did not fixate on the remaining 8. Later, when we ask you which billboards you recall, you might name 6, and when we combine the fixation and recall information, we may find out that these 6 are all from the group of 12 you had your eyes fixated on before. This is the gist of the procedure. The main challenge here is that the order of billboards was randomized across participants (to avoid serial position effects) and that different participants may look at completely different billboards. However, the strength is that this allows us to rigorously quantify whether actual exposure happened. We have revised the description as follows:

*Results:* … Next, we focused on the relationship between fixations on individual billboards and subsequent memory for the billboards across the two conditions. Toward this end, we determined for every participant the share of looked-at billboards (i.e., fixated at least once) that were later recalled (or recognized) and the corresponding share of billboards that were not-looked-at (i.e., never fixated). The resulting tabulation was combined with the information about the viewing condition and analyzed using ANOVA. Thus, the first factor varied between-subjects (20 participants in the free-viewing and 20 participants in the trash-counting condition), and the second factor was a within-subject factor defined on an individual basis according to participants’ viewing behavior and their subsequent memory performance (e.g., one participant could have recalled or recognized 5 billboards out of 10 fixated ones and 0 of the remaining 10 fixated ones; another participant could have recalled or recognized 8 out of 12 fixated ones and 1 out of the 8 fixated ones).

Regarding your question about how the participants were divided into the fixation categories, they were not divided into groups based on their fixation count. The variable fixation status was defined on an individual basis: Thus, for every participant, we know based on their eye-tracking which billboards they looked at and which ones they did not fixate. The resulting comparison is thus a repeated-measures/within-comparison and not a comparison between groups. This comparison also controls for individual differences in the degree to which people looked at billboards. Empirically, we do indeed find that some people showed a lot more fixations than others (from 30-150), which is due to factors like saccade frequency, arousal, or general curiosity. Statistically, the within-group comparison is the best approach to take care of this.

**R1.2b Additionally, the write-up mentions a resulting table in line 332, but I couldn't locate it.**

We apologize if our choice of the word “table” caused confusion, leading to the expectation that there was a table missing. This is not the case. Rather, what we intended to say was that we obtained for every participant two numbers - the number of looked-at billboards (i.e., fixated at least once) that were later recalled (or recognized) and the corresponding number of billboards that were not-looked-at (i.e., never fixated). As such, the word “table” simply referred to a “list of two numbers,” which was obtained for each participant. We have thus updated the language to remove this ambiguity. Additionally, the online reproducibility package does indeed contain the “full data table” that underlies the analysis. Thank you again for pointing out this potential source of misunderstanding.

*Results:* … Toward this end, we determined for every participant the number of looked-at billboards (i.e., fixated at least once) that were later recalled (or recognized) and the corresponding number of billboards that were not-looked-at (i.e., never fixated). The resulting tabulation was combined with the information about the viewing condition and analyzed using ANOVA.

**R1.2.c Furthermore, please rephrase line 352, which states that billboards that are never looked at are practically never recalled. If subjects can recall a billboard without seeing it, it would be considered blind guessing.**

We apologize if this wording led to confusion. We have rephrased this section.

*Results: …* Critically, however, the billboards that are never looked at are practically never brought up during the post-drive memory test.

Your comment about blind guessing is well-taken, and we agree that this is likely the most plausible option. However, there are at least two additional options that are scientifically possible and thus referred to in the discussion: 1) there could be parafoveal vision, i.e., participants could “see” even without properly fixating the billboards and therefore build some memory (see Calvo & Lang, 2005 article, which is mentioned in the discussion). 2) It could be that they only glance at them briefly without a full fixation (e.g., via ultra-rapid saccades, see Bacon-Mace et al., 2005, also cited in the discussion). The literature on eye-tracking and reading suggests that such ultra-rapid-vision does typically not result in much conceptual information processing, but the literature on vision suggests that even very brief glimpses as scenes (like the billboards visual gist) are enough to build memory (e.g. Schupp, Schmälzle, & Flaisch, 2014; Thorpe, 1996). In any case, your comment helped to make this clearer and avoid ambiguity, and we are grateful for this suggestion!

**R1.2.c … It is unclear why the authors chose to compare fixations between the two experimental conditions instead of conducting a correlation or regression analysis to link the number of exposures to recall.**

This comment again refers to the questions about analysis methods. As such, it has probably been clarified by our response to R1.2.a above, but for the sake of completeness, we provide a separate response here as well. The changes to the paper have already been listed above (see R1.2a.), and we thus refrain from repeating them here.

To avoid misunderstanding, which can easily happen when we refer to rather similarly sounding procedures, we briefly list the different analyses we ran that are relevant to the question:

- First, we compared the fixations between the two experimental conditions. The result (Figure 2, left panel) is that participants look at (i.e., fixate) the billboard far more often in the free viewing condition. Given that this manipulation (free-viewing vs. trash-counting) is a group variable, the appropriate statistical test for this is a t-test.
- Second, we combined the condition (free-viewing vs. trash-counting, a group-variable) with the viewing behavior (fixated billboard vs. not-fixated, defined individually and within-participants) in order to test how both variables affect the DV - recall (or recognition, respectively). The result (Figure 3) is that there are two main effects and an interaction: In the free viewing condition, participants remember more (main effect condition), and the billboards that are looked at are also remembered more (main effect of fixation status). Plus, there is an ordinal interaction such that fixated banners are remembered more in the free viewing condition.
- Third, we also attempted to “put the gradient in”, i.e., to resolve the variable “looked at” vs. “not looked at” in a more continuous way - by splitting the “looked at” category into a “looked at once” and “looked at more often” subgroups. The result (Figure 5) is that the higher the viewing behavior intensity, the better the memory. We had described this in our original submission as a dose-response relationship, but you had previously suggested we avoid this term, and we thus toned it down.
  Furthermore, the situation is such that the number of fixations is highly unevenly distributed: Many billboards are never fixated, many are fixated once and then abandoned, and multiple fixations (2,3,4 …) become progressively rarer. Thus, while one could argue that there is a continuum of viewing behavior intensity (as we also call it in Figure 5) that could support a regression analysis, the actual theoretical variable of interest is the difference between looked at vs. not-looked-at. This is a categorical variable, and thus ANOVA is the proper analysis (factoring in also the other manipulation, i.e., viewing condition). There are also some rather technical issues that arise regarding the eye-tracking parameter extraction (i.e., how to weigh in fixation length, consecutive or intermittent fixations, and so on), as well as the highly individualized analysis (i.e., different people looking at different billboards). We agree that these are all promising avenues for future research and this is also why we included the exploratory machine learning analysis, to which we turn next:
- Fourth, the machine-learning model (introduced as an exploratory analysis) further provides insight into the fixation-recall relationship, although this is a bit implicit in the way the model is set up. This is the case because the one-hot-encoded variable “banner identity” does implicitly “pick up” on the fixation information provided by the training data, i.e., the full information from participants’ fixation and memory behavior (across 40 participants and 20 banners, i.e., 800 training samples).
- Finally, we also report an exploratory correlation analysis regarding the number of fixations and recall at the by-participant level. We find that in the trash-counting condition, participants who looked more at billboards remembered them more, whereas in the free-viewing condition (in which participants generally looked at more billboards), there was no significant correlation between fixation count and memory.

Overall, while correlation and regression are important and valid statistical methods, they were not the ideal methods to examine the exact main research questions we were interested in, especially regarding the simple 2x2 question that is best assessed via an ANOVA. We hope that this clarifies all of your questions. Thank you again for giving us the opportunity to clarify the procedures.

Thank you very much again for the valuable suggestions and helpful comments.

***Reviewer #2:***

Reviewer 2 was satisfied with our revisions and had no further comments.
