# Supplementary material for: Examining the exposure-reception-retention link in realistic communication environments via VR and eye-tracking: The VR billboard paradigm
Source: PLoS One. 2023 Nov 30;18(11):e0291924. doi: 10.1371/journal.pone.0291924 (PMC10688884; doi:10.1371/journal.pone.0291924)
Supplement: Attachment — Submitted filename: ResponseToReviews_R3.docx [file pone.0291924.s004.docx]

***Response to reviews***

***Reviewer #1:***

**R1.1. : Thanks for the feedback. All looks good.**

We are pleased to hear that you found our revisions adequate and want to thank you again for the feedback and for the time and effort spent reviewing our manuscript! Below, we will again respond to your comments in a point-by-point fashion, again with excerpts of our changes to the manuscript in blue text.

**R1.2. I want to clarify one aspect of the ANOVA analysis. Is the dependent variable the memory performance/recall? With the current writing, I have some doubts about whether there are other complex mathematical calculations involved in defining the two groups. If the dependent variable is indeed recall, it might be better to remove the phrase "were later recalled or recognized" in line 351 and "subsequent memory performance" in line 356. Additionally, it might be more straightforward to state that recall is the dependent variable, rather than using a blanket example.. …**

Thank you for pointing out this potential source for confusion. Yes, the dependent variable is memory performance/recall and there are no complex calculations involved in defining the groups. We have removed the sections that you suggested; and we explicitly state that the dependent variable is recall. We also clarify that the same analysis was run on the recognition memory performance (rather than recall, but again without any complex calculations – just the same analysis as for recall).

*Line 309ff:* Note that the factor Fixation Status varies within subjects because every participant passed all 20 billboards and looked at some vs. ignored others. Viewing Condition represents a between subjects factor. In addition to computing this analysis using the recall data as the dependent variable, we also ran the same 2*2 ANOVA using the recognition memory as the dependent variable.

*Line 351 ff:* … Next, we focused on the relationship between fixations on individual billboards and subsequent memory for the billboards across the conditions. Toward this end, we determined for every participant the recall rate for looked-at billboards and the corresponding rate for not-looked-at-billboards.^3^ The resulting tabulation was combined with the information about the viewing condition and analyzed using ANOVA. Thus, the first factor (*Viewing Condition: free viewing vs. trash counting*) varied between-subjects (20 participants in the free-viewing and 20 participants in the trash-counting condition), and the second factor (*Fixation Status: looked-at vs. not-looked-at*) was a within-subject factor that was determined on an individual basis according to every participant’s viewing behavior. ^4^ The dependent variable in this 2*2 ANOVA was the free recall as our primary measure of memory performance. In addition to using free recall as the dependent variable, we also carried out the same 2*2-ANOVA for the recognition rate as another way of assessing subsequent memory.

Of note, we chose to provide information via two footnotes because we felt that this helps disrupting the flow of reading, but still gives the readers additional detail and explicit clarification in case they wonder about the specifics.

Footnote 3: recall_rate_looked-at-billboards_ = (number of billboards that were fixated at least once AND then recalled) / (number of billboards that were fixated at least once).

Footnote 4: For instance, one participant could have recalled 5 billboards out of 10 fixated ones (*recall_rate_looked-at-billboards_ =* 0.5) and 0 of the remaining 10 non-fixated ones, (*recall_rate_not-looked-at-billboards_ =* 0.0). By contrast, another participant could have recalled 8 out of 12 fixated ones and 1 out of the 8 non-fixated ones.

We agree that stating explicitly the dependent variable reduces ambiguity. More broadly, we thank you again for the valuable suggestions and helpful comments that have helped streamline the paper and make our message clearer.

***Reviewer #2:***

Reviewer 2 was satisfied with our prior revisions and had no further comments.
